# Brainstem white matter microstructure is associated with hyporesponsiveness and overall sensory features in autistic children

**DOI:** 10.1186/s13229-022-00524-3

**Published:** 2022-12-19

**Authors:** Olivia Surgent, Ali Riaz, Karla K. Ausderau, Nagesh Adluru, Gregory R. Kirk, Jose Guerrero-Gonzalez, Emily C. Skaletski, Steven R. Kecskemeti, Douglas C Dean III, Susan Ellis Weismer, Andrew L. Alexander, Brittany G. Travers

**Affiliations:** 1 Waisman Center, University of Wisconsin-Madison, 1500 Highland Avenue, Madison, WI 53705 USA; 2 Neuroscience Training Program, University of Wisconsin-Madison, Madison, WI USA; 3 Occupational Therapy Program in the Department of Kinesiology, University of Wisconsin-Madison, Madison, WI USA; 4 Department of Radiology, University of Wisconsin-Madison, Madison, WI USA; 5 Department of Medical Physics, University of Wisconsin-Madison, Madison, WI USA; 6 Department of Pediatrics, University of Wisconsin-Madison, Madison, WI USA; 7 Department of Communication Sciences and Disorders, University of Wisconsin-Madison, Madison, WI USA; 8 Department of Psychology, University of Wisconsin-Madison, Madison, WI USA; 9 Department of Educational Psychology, University of Wisconsin-Madison, Madison, WI USA; 10 Department of Psychiatry, University of Wisconsin-Madison, Madison, WI USA

**Keywords:** Brainstem, Sensory features, DTI, Autism, White matter, Voxel-based analysis

## Abstract

**Background:**

Elevated or reduced responses to sensory stimuli, known as sensory features, are common in autistic individuals and often impact quality of life. Little is known about the neurobiological basis of sensory features in autistic children. However, the brainstem may offer critical insights as it has been associated with both basic sensory processing and core features of autism.

**Methods:**

Diffusion-weighted imaging (DWI) and parent-report of sensory features were acquired from 133 children (61 autistic children with and 72 non-autistic children, 6–11 years-old). Leveraging novel DWI processing techniques, we investigated the relationship between sensory features and white matter microstructure properties (free-water-elimination-corrected fractional anisotropy [FA] and mean diffusivity [MD]) in precisely delineated brainstem white matter tracts. Follow-up analyses assessed relationships between microstructure and sensory response patterns/modalities and analyzed whole brain white matter using voxel-based analysis.

**Results:**

Results revealed distinct relationships between brainstem microstructure and sensory features in autistic children compared to non-autistic children. In autistic children, more prominent sensory features were generally associated with lower MD. Further, in autistic children, sensory hyporesponsiveness and tactile responsivity were strongly associated with white matter microstructure in nearly all brainstem tracts. Follow-up voxel-based analyses confirmed that these relationships were more prominent in the brainstem/cerebellum, with additional sensory-brain findings in the autistic group in the white matter of the primary motor and somatosensory cortices, the occipital lobe, the inferior parietal lobe, and the thalamic projections.

**Limitations:**

All participants communicated via spoken language and acclimated to the sensory environment of an MRI session, which should be considered when assessing the generalizability of this work to the whole of the autism spectrum.

**Conclusions:**

These findings suggest unique brainstem white matter contributions to sensory features in autistic children compared to non-autistic children. The brainstem correlates of sensory features underscore the potential reflex-like nature of behavioral responses to sensory stimuli in autism and have implications for how we conceptualize and address sensory features in autistic populations.

**Supplementary Information:**

The online version contains supplementary material available at 10.1186/s13229-022-00524-3.

## Background

Approximately 90% of autistic children [1, 2] and 5–15% of non-autistic children exhibit elevated sensory features, characterized by enhanced or reduced reactivity to or interest in sensory stimuli [3–5]. (Please note, identity-first language is used in alignment with the majority preference of the autistic community [6, 7].) These elevated sensory features are associated with decreased motor performance [8], increased core autism traits [9, 10], increased anxiety [11, 12], decreased adaptive behaviors [13], and decreased quality of life [14, 15]. Currently, the neurobiological mechanisms of sensory features in autistic and non-autistic populations are unclear [16]. However, the underexplored brainstem may offer critical insights into the neural basis of sensory features due to its established role in sensory processing [17, 18] and associations with core autism traits [19, 20]. Therefore, the purpose of the present study was to examine the relationship between brainstem microstructure and sensory features in autistic and non-autistic children.

The brainstem is an early developing and highly conserved [21] structure that is comprised of tightly intertwined white matter tracts, many of which have been linked to sensory processing. Brainstem white matter fibers serve as initial conduits of sensory information, relaying signals from primary sensory organs to nuclei within the brainstem, cerebrum, and cerebellum [17, 22–24]. Brainstem white matter tracts further support basic sensory information processing by transmitting sensory signals among nuclei with demonstrated roles in sensory gating [25, 26], visual salience [27], multisensory integration [28], and sensory responsivity [29, 30]. While much of this work has been done in animal models, similar associations in humans have been established between brainstem white matter and the early stages of sensory processing [26, 31–33]. Moreover, early developing brainstem pathways are known to subserve early-in-life auditory, visual, gustatory, olfactory processing as well as tactile-motor integration (as reviewed by [18]). However, it remains unclear how brainstem white matter is related to sensory responses in autism. Despite the brainstem’s demonstrated role in the fundamental elements of sensory processing, previous work looking at the neural contributions to sensory response patterns has largely focused on telencephalic structures as key regions of interest [34–39]. Therefore, we still do not know whether brainstem white matter contributions are limited to relaying and processing basic sensory information or extend into producing heightened or reduced sensory responses.

Evaluating sensory features and their relationships to brainstem microstructure in autistic populations is critical as evidence indicates that brainstem white matter may uniquely contribute to autism [19] and elevated sensory features are highly prevalent in the autistic population. Epidemiological, molecular, and behavioral evidence suggests that brainstem organization may be closely tied to the etiology of autism [18–20, 40]. Indeed, one of the earliest hypotheses regarding the neural basis of autism centered upon the brainstem’s reticular formation [41]. More recently, several articles have reviewed the evidence of the brainstem’s role in autism and have put forth theories about how the structure, function, and development of brainstem white matter tracts and gray matter nuclei may be involved in autistic traits [18–20]. Additionally, an exploratory analysis from Wolff and colleagues [42] linked sensory features to brainstem-cerebellar white matter, finding that infants who later received an autism spectrum diagnosis showed inverse sensory-microstructure correlations compared to infants who did not receive a diagnosis. These diagnosis-dependent neural correlates of sensory features in autism are supported by evidence suggesting the presentation of sensory features and their neurobiological bases may be unique in autism compared to non-autism and/or other psychiatric conditions [43]. For example, evidence suggests that sensory hyporesponsiveness in autistic populations may be unique in both its behavioral presentation and neural basis. Hyporesponsiveness is more prevalent in autistic individuals than in other populations [36, 44–47] and has been associated with altered patterns of neural activity in infants with and without a predisposition for autism [48]. This evidence coupled with the distinct contributions of the brainstem to autism traits [19] highlights the need for a direct comparison of brainstem neural correlates in autistic and non-autistic youth. This direct comparison will determine not only how the brainstem is involved in sensory processing but also if its involvement is similar or distinct in autistic and non-autistic populations. Previously, methodological constraints limited the feasibility of collecting high resolution diffusion-weighted imaging (DWI) data (traditionally a time consuming and sensory intensive process) in pediatric populations with sensory features. However, recent advancements in our DWI protocol have allowed us to overcome these limitations, providing high apparent resolution and improved gray-white matter contrast without requiring long acquisition times [49]. These innovations offer the opportunity to investigate white matter microstructure of brainstem tracts in children with elevated sensory features with a higher degree of precision than ever before.

Using our optimized DWI, the aim of this study was to determine the extent to which brainstem white matter tracts are associated with individual differences in the sensory features of autistic and non-autistic children (6–11 years of age). Even though the brainstem begins to form in the first trimester of pregnancy [50], there is evidence that the brainstem tracts subserving vision undergo activity-dependent myelination based on sensory stimulation in the first year of life [51]. Auditory, olfactory, tactile, and gustatory brainstem tracts are likely to similarly undergo post-natal tuning based on sensorimotor experiences [18, 20]. This experience-based tuning may lead to cascading white matter differences in school-aged autistic children and beyond. Therefore, this age range was selected for feasibility of collecting the MRI parameters and with the idea that differences in early-maturing brainstem circuits may continue to subserve the sensory features commonly reported in autistic children [18]. Based on literature from animal models and humans involving brainstem white matter in basic sensory processing, we hypothesized that brainstem microstructure as measured by diffusion MRI (free-water-eliminated fractional anisotropy [FWE-FA] and mean diffusivity [FWE-MD]) would be related to the presence of elevated sensory features in both autistic and non-autistic children. While other DTI measures are possible to calculate, we chose FWE-FA and FWE-MD based on FA and MD findings of previous literature [42] and evidence of reduced artifacts in brain areas surrounded by cerebrospinal fluid (CSF) when using FWE [52, 53]. While FWE-DTI measures do not directly measure microstructure, they are commonly used as markers sensitive to changes in white matter microstructural features, including axonal morphology and myelination, axon bundle density and fiber orientation distribution, and other intra- and extra- cellular processes. Based on the evidence suggesting unique brainstem involvement in autism [19] and a diagnosis-dependent relationship between sensory features and white matter microstructure [42], we further examined the possibility that sensory-brainstem relationships would be unique within each diagnostic group. To test these hypotheses, we performed region of interest (ROI) linear regression predicting FWE-FA and FWE-MD of brainstem tracts from sensory caregiver report and diagnostic group status, while controlling for key variables such as age, sex, and head motion during the DWI scan. A significant main effect for sensory features would support that the FWE-FA and FWE-MD of the brainstem white matter tracts are significantly associated with caregiver-reported sensory features across groups. A significant group-by-sensory interaction would support our hypothesis of unique brainstem-sensory relations in autistic compared to non-autistic children. Follow-up analyses explored these effects within the autistic group as a function of sensory pattern and sensory modality. To contextualize brainstem findings, follow-up, whole-brain voxel-based correlates of sensory features were assessed across both groups and within just the autistic group.

## Methods

### Participants

156 participants were enrolled and participated in this study. However, as can be seen in Additional file 1: Fig. 1, due to a scanner upgrade malfunction that affected scans (*n* = 8), incomplete DWI data (*n* = 10), incomplete T1-weighted [T1w] structural data (*n* = 1), DWI scans not meeting our quality control standards (*n* = 3), and an extreme outlier in the SEQ behavioral data (*n* = 1), the final sample was 133 participants (ages 6.0–10.9, 37 female), with 61 in the autistic diagnostic group (6.14–10.90 years, 12 female) and 72 in the non-autistic group (6.02–10.97 years, 25 female). A very conservative a priori power analysis was conducted to determine our ability to detect voxel-based findings (Additional File 1). Due to COVID-19, the autistic group's sample size was below the intended sample size of the power analysis. All participants were required to communicate verbally and have an IQ score greater than 60 using the Wechsler Abbreviated Scale of Intelligence, 2nd Edition (WASI-2) [54] or greater than 70 on the Kaufman Brief Intelligence Test-Second Edition (KBIT-2) [55]. None of the participants had a previous diagnosis of tuberous sclerosis, Down syndrome, fragile X, hypoxia–ischemia, notable and uncorrected hearing or vision loss, or a history of severe head injury. The institutional review board at the University of Wisconsin–Madison approved all procedures. In each case, the child participant provided assent and a parent or guardian provided informed consent.

To confirm previous community diagnoses of autism spectrum disorder (ASD), participants in the autistic group were comprehensively evaluated for ASD by meeting cutoffs on either (1) the Autism Diagnostic Observation Schedule, 2nd edition (ADOS-2; cutoff = 8) [56] and the Autism Diagnostic Interview-Revised (ADI-R) [57] or (2) the Social Responsiveness Scale, second edition (SRS-2; cutoff = 60) [58] and the Social Communication Questionnaire (SCQ; cutoff = 15) [59].

Non-autistic participants were required to score less than 8 on the SCQ [59]. Additionally, participants were excluded from the non-autistic group if they had a previous diagnosis of any neurodevelopmental disorder including attention deficit/hyperactivity disorder, bipolar disorder, major depressive disorder, or if they had a first-degree relative with ASD. Table 1 contains group contrasts in the demographic information.

**Table 1:** Demographic information for participant sample

	Autistic (*n* = 61)	Non-Autistic (*n* = 72)	*t*	*p*
Age(years), Mean(SD)	8.65(1.33)	8.28(1.38)	− 1.5	.13
Sex, % Female	20%	35%	–	–
Average Head Motion (AVD), Mean(SD)	0.63(0.44)	0.52(0.41)	− 1.5	.13
IQ, Standardized Score, Mean(SD)	103(18)	114(12)	4.34	< .001
SCQ, Mean(SD)	20.16(6.6)	1.43(1.95)	− 22.96	< .001
SEQ 3.0 Total Sensory Features, Mean(SD)	2.36(0.44)	1.52(0.23)	− 14.18	< .001
SEQ 3.0 Hyporesponsiveness, Mean(SD)	1.99(0.50)	1.27(0.20)	− 11.17	< .001
SEQ 3.0 Hyperresponsiveness, Mean(SD)	2.66(0.54)	1.44(0.32)	− 16.06	< .001
SEQ 3.0 Enhanced Perception, Mean(SD)	2.42(0.63)	1.84(0.42)	− 6.38	< .001
SEQ 3.0 Sensory Seeking, Mean(SD)	2.38(0.67)	1.53(0.31)	− 9.70	< .001
SEQ 3.0 Visual Sensitivity, Mean(SD)	2.35(0.62)	1.42(0.28)	− 11.31	< .001
SEQ 3.0 Auditory Sensitivity, Mean(SD)	2.40(0.56)	1.58(0.35)	-9.86	< .001
SEQ 3.0 Tactile Sensitivity, Mean(SD)	2.48(0.49)	1.53(0.31)	− 12.99	< .001
SEQ 3.0 Gustatory Sensitivity, Mean(SD)	2.55(0.72)	1.53(0.38)	− 10.32	< .001
SEQ 3.0 Vestibular Sensitivity, Mean(SD)	2.25(0.54)	1.58(0.33)	− 8.56	< .001

*AVD* Average volume displacement [60], *SCQ* Social communication questionnaire [59], *SEQ* 3.0 Sensory experience questionnaire, Version 3.0 [61]; Standardized IQ scores are harmonized across participants who completed the Kaufman Brief Intelligence Test, Second Edition (KBIT-2; Autistic *n* = 16, Non-Autistic *n* = 20) [55] or the Wechsler Abbreviated Scale of Intelligence, Second Edition (WASI-II; Autistic *n* = 45, Non-Autistic *n* = 52) [54]

### Sensory experience questionnaire version 3.0 (SEQ 3.0)

The SEQ 3.0 is a 105-item caregiver report instrument developed to characterize sensory features in both non-autistic individuals and in those with certain developmental disorders, including ASD. The assessment is designed for use in children aged 2–12 years-old and measures sensory responses to experiences using a 5-point scale, with higher scores representing more prominent sensory features [61]. The questionnaire contains 97 items which specifically measure the occurrence of behaviors across sensory response patterns (hyperresponsiveness, hyporesponsiveness, enhanced perception, sensory seeking) and across sensory modalities (visual, auditory, gustatory, tactile, and vestibular). By combining the results from items, a composite score of overall sensory features can be calculated. A subset of these SEQ 3.0 data have been previously used to examine associations among sensory features, motor skills, and IQ [8].

### Brain imaging acquisition and processing

Imaging data were acquired on a 3T GE Discovery MR750 scanner (Waukesha, WI) in the Waisman Center at the University of Wisconsin–Madison. Diffusion-weighted images (DWIs) were obtained using a 32-channel phased array head coil (Nova Medical, Wilmington, MA) and a multi-shell spin-echo echo-planar imaging (EPI) pulse sequence (9 directions at b = 350 s/mm^2^, 18 directions at 800 s/mm^2^, and 36 directions at b = 2000s/mm^2^, and 6 non-diffusion-weighted [b = 0 s/mm^2^] volumes; TR/TE = 9000/74.4 ms; FOV = 230 mm × 230 mm, in-plane resolution 2.4 mm × 2.4 mm, interpolated to 1.8 mm × 1.8 mm; 76 overlapping slices, slice thickness 3.6 mm, spacing between slice centers 1.8 mm – to achieve 1.8 mm isotropic sampling). An additional 6 non-diffusion-weighted volumes with reverse phase-encoded direction were collected for use in correcting susceptibility-induced artifacts [62], which may be severe around the brainstem in EPI acquisitions and affect interpretability of data in these regions. The approximate duration of the DWI scan was 10 min. Whole-brain structural imaging was done using a 3D T1w MPnRAGE sequence with 1 mm isotropic resolution (approximately 8 min). The MPnRAGE pulse sequence is a novel imaging method that combines magnetization preparation using inversion recovery with a rapid 3D radial k-space readout [63]. The MPnRAGE reconstruction enables retrospective head-motion correction, tissue-specific segmentation, and reliable quantitative T1 mapping [64].

DWI data were processed to minimize noise [65, 66], Gibbs ringing [67], artifacts caused by motion, eddy current [68–70], EPI distortion [62], as well as B0 field inhomogeneities [71, 72]. To enhance the apparent spatial resolution, DWI data were then processed in accordance to TiDi-Fused protocol [49]. The mean DWI b = 0 volume was spatially aligned to the T1 weighted image derived from the MPnRAGE using rigid transformations (6 degrees of freedom) implemented with the boundary-based registration (BBR) [73] routine in the FreeSurfer image analysis suite [74]. The estimated transformation that resulted from the optimal alignment was then applied to the entire DWI series with cubic B-spline interpolation up-sampled to the T1w resolution (1 mm isotropic) using ANTs [75]. The rotational component of the rigid body transformation was then applied to the DWI encoding directions.

Free water eliminated (FWE) diffusion tensor imaging (DTI), which has been shown to produce more complete, anatomically plausible tract reconstructions in regions with suspected CSF partial volume artifacts [76], was used during diffusion tensor estimation. FWE fractional anisotropy (FWE-FA) and FWE mean diffusivity (FWE-MD) maps were generated from the resulting tensor maps [74, 77]. FWE-DTI metrics are sensitive to changes in in vivo tissue microstructural properties, particularly the density and organization of axons in white matter. Increased FWE-FA and decreased FWE-MD are commonly associated with more dense and more organized white matter tracts. The average relative voxel displacement between volumes acquired during the DWI scan was estimated using *eddy_qc* and utilized to quantify participant head motion [78]. All FWE-DTI images passed a visual inspection for processing artifacts prior to statistical analyses.

## Statistical analysis

### Brainstem white matter region of interest analysis

Since the regions were based on probabilistic tractography visitation counts (normalized to values between 0 and 1 at each voxel), we computed summary diffusion measures in each bundle using the weighted median. Weighted median [79] values of the FWE-DTI measures were extracted from 23 bilateral brainstem fiber bundles (Additional file 1: Fig. 2) defined on a probabilistic brainstem connectome atlas [80]. Using ‘antsRegistration’ [75] with affine and diffeomorphic transformations, tracts were warped to a T1w study specific template that was aligned with the MNI152 T1w image. The tracts were then mapped to subject specific native space by applying the inverse transformations estimated during the template construction. The tract representations were inspected visually to ensure a faithful representation of the spatial pattern and anatomical placement were obtained. Inspection of the tract representations showed that the superior cerebellar peduncle-cerebellorubral (SCPCR) tract was fully encompassed by the superior cerebellar peduncle-cerebellothalamic (SCPCT) tract. Therefore, the final analyses included 11 regions of interest (ROIs; 10 bilateral bundles and the middle cerebellar peduncle [MCP] bundle) and excluded the SCPCR. Bundles were warped to the native subject space, with cubic interpolation, using the transforms generated during population template estimation. While some evidence from functional neuroimaging studies suggests cerebellar asymmetry and asymmetry in brainstem based auditory processing [81, 82], there is little current evidence to suggest global structural asymmetry in brainstem or cerebellar white matter tracts [83]. Therefore, primary analyses utilized the bilateral average of the weighted median from each bundle. However, follow-up analyses were conducted to evaluate potential laterality effects. All bundles were quality assessed by outlier analysis of the summary measures. Tracts that demonstrated significant relationships between FWE-DTI and sensory features after multiple comparison correction additionally passed a visual inspection performed in each DWI scan’s native space. FWE-DTI metrics did not significantly differ between the autistic and non-autistic groups in any of the white matter bundles (Additional file 1: Table 1).

Using multiple linear regression, FWE-DTI metrics (FA and MD) in each of the 11 brainstem ROIs were predicted as a function of diagnostic group (autistic vs non-autistic), overall sensory features, and their interaction, while controlling for age, sex, and head motion. Using partial Pearson correlations (controlling for age, sex, and head motion), follow-up ROI analyses were conducted within the autistic group examining FWE-DTI metrics of each bilateral bundle in relation to each sensory response pattern and modality [84]. FDR multiple comparison correction was employed across the 11 bundles for each FWE-DTI metric (FDR-adjusted *p* < 0.05) [85].

Follow-up analyses investigated the relationship between sensory features and brainstem white matter metrics (FA and MD) estimated using traditional tensor fitting algorithms [86] without FWE correction. Additional follow-up analyses were performed using a linear mixed effects models to examine the effects of white matter tract laterality on group-by-overall sensory feature interaction effects. These analyses predicted FWE-DTI of each tract from diagnostic group, overall sensory features, tract laterality (right vs left), each of their two-way interactions, and their three-way interaction while controlling for age, sex, and head motion and including a random effect for participant.

### Follow-up voxel-based analysis with tissue-specific, smoothing-compensated (T-SPOON)

To investigate whether the diagnosis-dependent relationships found between FWE-MD and sensory features were specific to brainstem white matter or reflective of an altered whole-brain white-matter system, we performed tissue-specific, smoothing compensated (T-SPOON) method for voxel-based analysis (VBA). T-SPOON was implemented to account for common pitfalls of traditional VBA and to enhance the interpretability of VBA results [87]. T-SPOON-corrected FWE-MD maps were generated in accordance with previous work [88]. T-SPOON VBA was utilized rather than other whole brain analysis techniques, such as tract based spatial statistics (TBSS [89]), as it allows for a more accurate representation of brainstem white matter tracts. In fact, an in-house test suggested that the TBSS skeleton only represented 21% of brainstem white matter. Permutation Analysis of Linear Models (PALM) was used to perform voxel-wise statistical parametric mapping [90–92]. Using linear regression, FWE-MD was predicted as a function of overall sensory features, diagnosis (autistic/non-autistic), and their interaction while accounting for age, sex, and average head motion. A follow-up VBA was performed to identify areas where FWE-MD was associated with overall sensory features within the autistic group. For all VBAs, multiple comparisons correction was performed using FDR-correction (FDR adjusted *p* < 0.05) [85, 90].

## Results

### ROI brainstem results for sensory main effects and sensory-by-diagnosis interactions

**Fig. 1:**
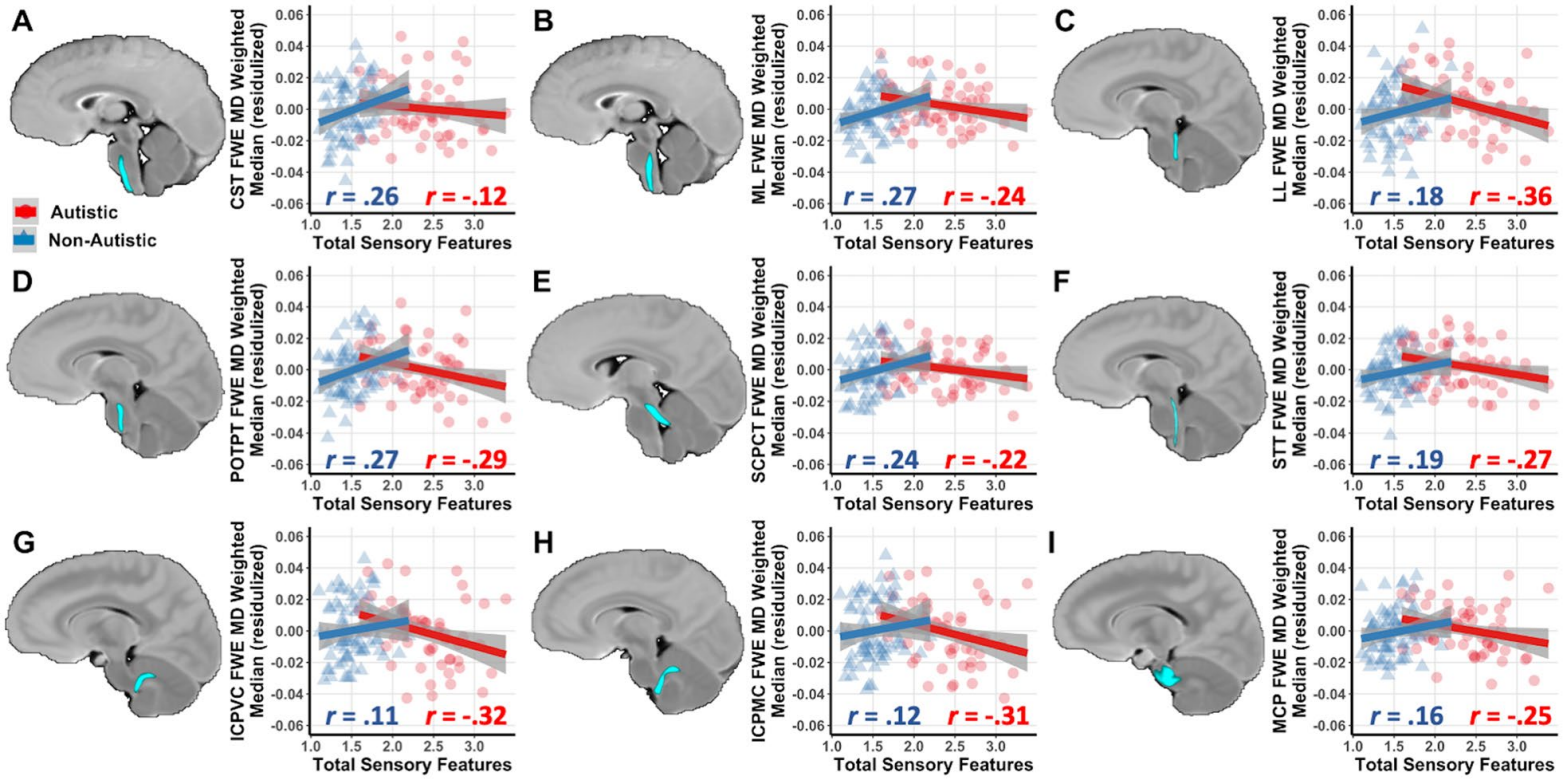
Diagnosis-dependent relationships between brainstem white matter microstructure and total sensory features. Brainstem white matter tracts that exhibit total sensory feature-by-diagnosis interaction effects for the free-water-elimination mean diffusivity (FWE-MD). Analyses account for age, sex, and average head motion and apply an FDR correction for multiple comparisons. Correlations within the autistic (red circles) and non-autistic (blue triangles) groups are shown in the **A** corticospinal tract (CST), **B** medial lemniscus (ML), **C** lateral lemniscus (LL), **D** parieto-occipito-temporo-pontine tract (POTPT), **E** superior cerebellar peduncle cerebellothalamic tract (SCPCT), **F** spinothalamic tract (STT), **G** inferior cerebellar peduncle vestibulocerebellar tract (ICPVC), **H** inferior cerebellar peduncle medulla-cerebellar tract (ICPMC), and **I** middle cerebellar peduncle (MCP)

We examined FWE-FA and FWE-MD in each brainstem white matter tract as a function of total sensory features and their interaction with diagnosis (autistic vs non-autistic). No significant main effects were found in models predicting FWE-MD or FWE-FA. Significant sensory features-by-diagnosis interaction effects were found for FWE-MD in the following tracts: corticospinal tract (CST), medial lemniscus (ML), lateral lemniscus (LL), parieto-occipito-temporo-pontine tract (POTPT), spinothalamic tract (STT), superior cerebellar peduncle cerebellothalamic tract (SCPCT), inferior cerebellar peduncle vestibulocerebellar tract (ICPVC), inferior cerebellar peduncle medulla-cerebellar tract (ICPMC), and middle cerebellar peduncle (MCP). Elevated total sensory features were associated with decreased FWE-MD in the autistic group and increased FWE-MD in the non-autistic group (*p* < 0.05, FDR-corrected) (Fig. 1, Table 2). No significant interaction effects were found for FWE-FA after FDR correction (Table 3). Follow-up analyses sought to determine if effects were lateralized to right or left brainstem white matter pathways but found no significant effects after FDR correction (Additional file 1: Table 2). Together, these findings indicate a diagnosis-dependent relationship between total sensory features and brainstem white matter microstructure, specifically FWE-MD.

**Table 2:** Effects of total sensory features on brainstem FWE-MD in autistic and non-autistic children

Brainstem White Matter Region	Total sensory features main effect				Group x total sensory Features interaction effect			
	b	Std Error	t	p	b	Std Error	t	p
CST	0.008	0.005	1.51	.13	− 0.026	0.011	− 2.41	.02^a,b^
ML	0.004	0.004	1.06	.29	− 0.025	0.008	− 3.07	< .001 ^a,b^
LL	− 0.001	0.005	− 0.12	.90	− 0.032	0.011	− 2.93	< .001 ^a,b^
FPT	0.007	0.007	0.94	.35	− 0.019	0.014	− 1.31	.19
POTPT	0.004	0.005	0.93	.36	− 0.032	0.010	− 3.25	< .001 ^a,b^
STT	0.001	0.004	0.36	.72	− 0.020	0.008	− 2.51	.01 ^a,b^
SCPCT	0.004	0.004	1.10	.27	− 0.022	0.008	− 2.77	.01 ^a,b^
SCPSC	0.002	0.006	0.33	.74	− 0.011	0.012	− 0.95	.34
ICPMC	− 0.002	0.006	− 0.28	.78	− 0.025	0.012	− 2.14	.03 ^a,b^
ICPVC	− 0.002	0.006	− 0.40	.69	− 0.025	0.012	− 2.15	.03 ^a,b^
MCP	0.001	0.004	0.20	.85	− 0.020	0.009	− 2.22	.03 ^a,b^

Main and interaction effects controlling for age, sex and average head motion during DWI brain scan

ML medial lemniscus; LL lateral lemniscus; STT spinothalamic tract; SCPCT superior cerebellar peduncle cerebellothalamic tract; SCPSC superior cerebellar peduncle spinocerebellar tract; MCP middle cerebellar peduncle; ICPMC inferior cerebellar peduncle tracts from medulla oblongata to the cerebellum; ICPVCT inferior cerebellar peduncle vestibulocerebellar tract; FPT frontopontine tract; POTPT parieto-occipito-temporo-pontine tract; CST corticospinal tract

^a^Uncorrected *p* < .05

^b^False discovery rate (FDR) corrected *p* < .05

**Table 3:** Effects of total sensory features on brainstem FWE-FA in autistic and non-autistic children

Brainstem white matter region	Total sensory features main effect				Group x total sensory features interaction effect			
	*b*	Std Error	*t*	*p*	*b*	Std Error	*t*	*p*
CST	0.002	0.011	0.13	.89	0.009	0.023	0.38	.71
ML	− 0.008	0.008	− 1.02	.31	0.042	0.016	2.56	.01^a^
LL	− 0.005	0.013	− 0.36	.72	0.056	0.027	2.07	.04^a^
FPT	− 0.016	0.012	− 1.30	.19	0.023	0.026	0.91	.37
POTPT	− 0.013	0.009	− 1.38	.17	0.028	0.019	1.48	.14
STT	0.001	0.009	0.14	.89	0.011	0.018	0.63	.53
SCPCT	− 0.001	0.006	− 0.09	.93	0.015	0.013	1.12	.26
SCPSC	− 0.012	0.010	− 1.19	.23	0.015	0.021	0.74	.46
ICPMC	0.000	0.009	0.05	.96	0.023	0.019	1.18	.24
ICPVC	0.006	0.010	0.55	.58	0.019	0.021	0.91	.36
MCP	0.013	0.008	1.62	.11	0.026	0.017	1.55	.12

Main and interaction effects controlling for age, sex and average head motion during DWI brain scan

ML medial lemniscus; LL lateral lemniscus; STT spinothalamic tract; SCPCT superior cerebellar peduncle cerebellothalamic tract; SCPSC superior cerebellar peduncle spinocerebellar tract; *MCP* Middle cerebellar peduncle, *ICPMC* Inferior cerebellar peduncle tracts from medulla oblongata to the cerebellum, *ICPVCT* Inferior cerebellar peduncle vestibulocerebellar tract, *FPT* Frontopontine tract, *POTPT* Parieto-occipito-temporo-pontine tract, *CST* Corticospinal tract

^a^Uncorrected *p* < .05

^b^False discovery rate (FDR) corrected *p* < .05

### Follow-Up ROI brainstem results for sensory response patterns within the autistic group

**Fig. 2:**
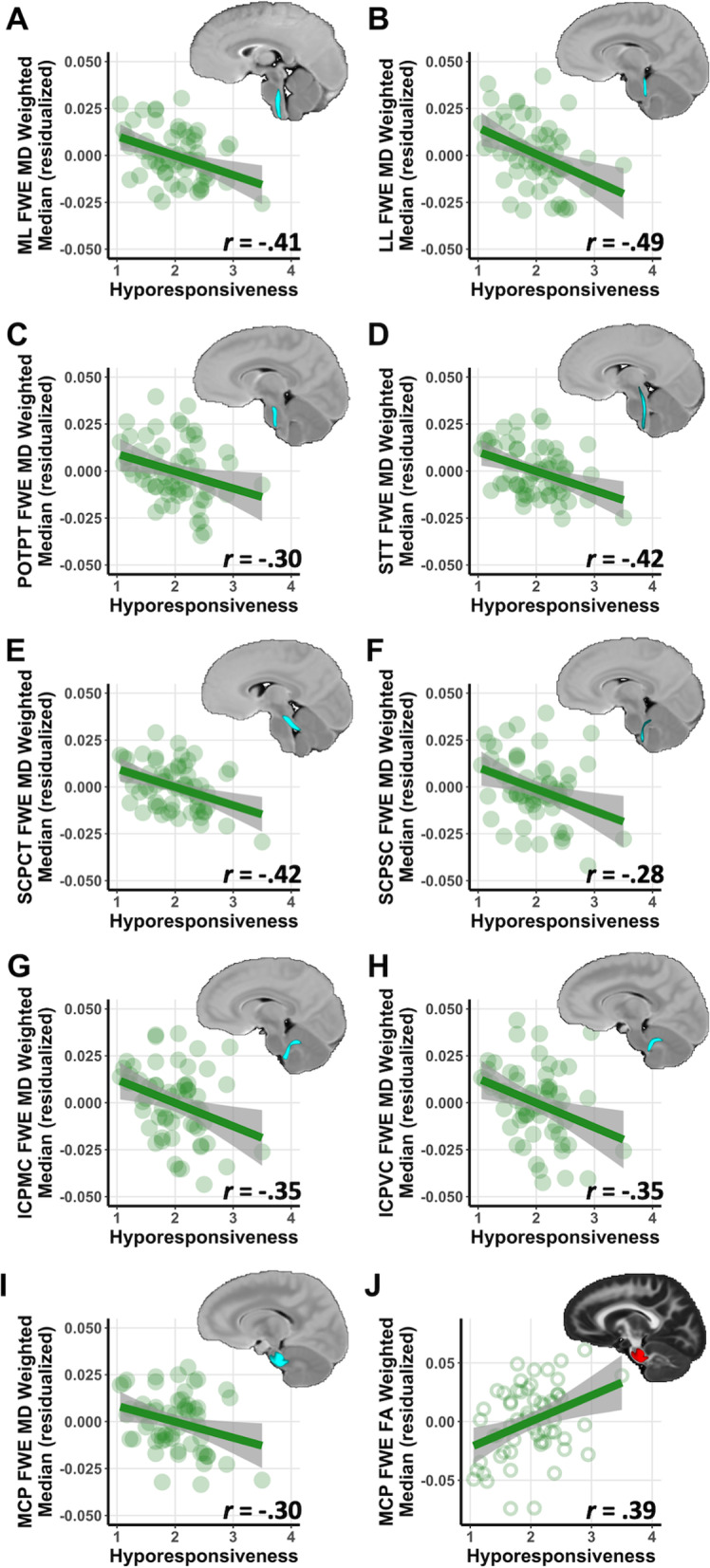
Correlations between hyporesponsiveness and brainstem white matter microstructure in autistic children. Brainstem white matter tracts showed significant relationships between hyporesponsiveness and microstructural properties after accounting for age, sex, and average head motion and applying an FDR correction for multiple comparisons. Significant correlations were found with free-water-elimination mean diffusivity (FWE-MD) in the **A** medial lemniscus (ML), **B** lateral lemniscus (LL), **C** parieto-occipito-temporo-pontine tract (POTPT), **D** spinothalamic tract (STT), **E** superior cerebellar peduncle cerebellothalamic tract (SCPCT), **F** superior cerebellar peduncle spinocerebellar tract (SCPSC), **G** inferior cerebellar peduncle medulla-cerebellar tract (ICPMC), **H** inferior cerebellar peduncle vestibulocerebellar tract (ICPVC), **I** middle cerebellar peduncle (MCP), and **J** FWE-FA in the MCP

Within the autistic group, follow-up analyses were conducted to assess the relationship between FWE-DTI measures and specific sensory response patterns (Table 4). Hyporesponsiveness was negatively associated with FWE-MD in nine of 11 brainstem tracts and was positively associated with FWE-FA in the MCP (*p* < 0.05, FDR-corrected) (Fig. 2). There were no significant associations for hyperresponsiveness, sensory seeking, nor enhanced perception after FDR correction (Table 4).

**Table 4:** Brainstem white matter regions of interest and sensory response patterns in the autistic group

Brainstem white matter region	Hyporesponsiveness		Hyperresponsiveness		Enhanced perception		Sensory seeking	
	FWE-FA	FWE-MD	FWE-FA	FWE-MD	FWE-FA	FWE-MD	FWE-FA	FWE-MD
	*r*	*r*	*r*	*r*	*r*	*r*	*r*	*r*
CST	.30 ^a^	− .22	.06	− .11	− .08	− .05	.06	− .14
ML	.23	− .41 ^a,b^	.09	− .25	.07	− .20	.23	− .15
LL	.18	− .49 ^a,b^	.08	− .21	.15	− .21	.20	− .33^a^
FPT	.12	− .04	− .17	− .09	− .11	− .06	.08	− .13
POTPT	.15	− .30 ^a,b^	− .03	− .19	− .05	− .23	− .07	− .29 ^a^
STT	.10	− .42 ^a,b^	.07	− .25	< .001	− .25	.17	− .19
SCPCT	.20	− .42 ^a,b^	.07	− .12	− .01	− .11	.18	− .17
SCPSC	.02	− .28 ^a,b^	− .08	− .07	− .11	− .03	.06	− .09
ICPMC	.18	− .35 ^a,b^	.06	− .24	.14	− .20	.08	− .21
ICPVC	.16	− .35 ^a,b^	.11	− .23	.14	− .20	.09	− .22
MCP	.39 ^a,b^	− .30 ^a,b^	.37^a^	− .21	.25	− .18	.27 ^a^	− .20

Partial correlations account for age, sex and average head motion during DWI brain scan

*CST* Corticospinal tract, *ML* Medial lemniscus, *LL* Lateral lemniscus, *STT* Spinothalamic tract, *SCPCT* Superior cerebellar peduncle cerebellothalamic tract, *SCPSC* Superior cerebellar peduncle spinocerebellar tract, *MCP* Middle cerebellar peduncle, *ICPMC* Inferior cerebellar peduncle tracts from medulla oblongata to the cerebellum, *ICPVCT* Inferior cerebellar peduncle vestibulocerebellar tract, *FPT* Frontopontine tract, *POTPT* Parieto-occipito-temporo-pontine tract

^a^Uncorrected *p* < .05

^b^False discovery rate corrected (FDR) *p* < .05

### Follow-Up ROI brainstem results for sensory modalities within the autistic group

**Fig. 3:**
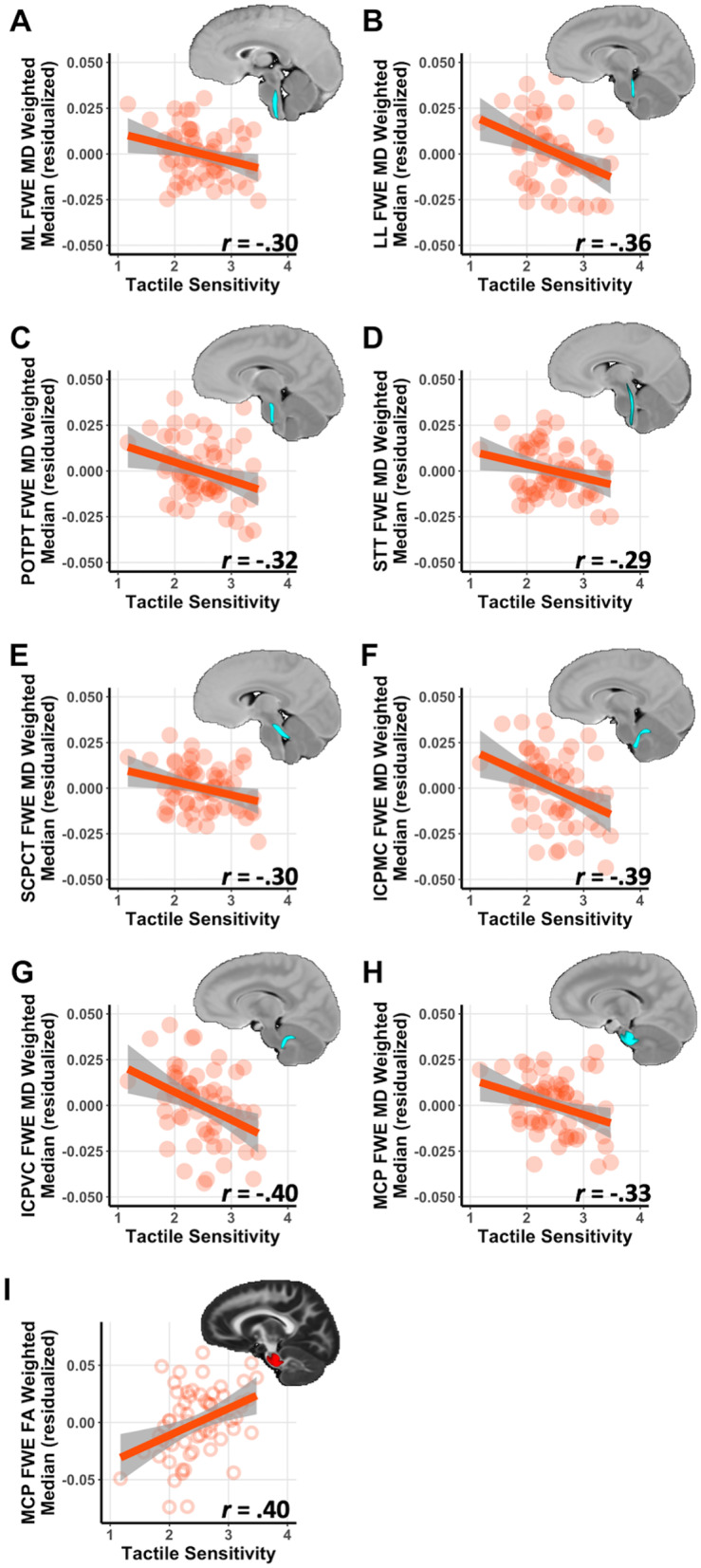
Correlations between tactile sensitivity and brainstem white matter microstructure in autistic children. Brainstem white matter tracts showed significant relationships between tactile sensitivity and microstructural properties after accounting for age, sex, and average head motion and applying an FDR correction for multiple comparisons. Significant correlations were found with free-water-elimination mean diffusivity (FWE-MD) in the **A** medial lemniscus (ML), **B** lateral lemniscus (LL), **C** parieto-occipito-temporo-pontine tract (POTPT), **D** spinothalamic tract (STT), **E** superior cerebellar peduncle cerebellothalamic tract (SCPCT), **F** inferior cerebellar peduncle medulla-cerebellar tract (ICPMC), **G** inferior cerebellar peduncle vestibulocerebellar tract (ICPVC), **H** middle cerebellar peduncle (MCP), and **I** FWE-FA in the MCP

Within the autistic group, additional follow-up analyses were conducted to assess the relationship between FWE-DTI measures and sensory modalities. Tactile sensitivities were associated with FWE-MD in eight of 11 tracts and FWE-FA in the MCP (Fig. 3, Table 5). Further, visual sensitivities were associated with FWE-MD in the LL and POTPT, gustatory sensitivities were associated with FWE-FA in the MCP, and vestibular sensitivities were associated with FWE-MD in the LL. No FWE-DTI correlations were found with auditory sensitivities (Table 5).

**Table 5:** Brainstem white matter regions of interest and sensory modalities in the autistic group

Brainstem White Matter Region	Visual		Tactile		Gustatory		Auditory		Vestibular	
	FWE-FA	FWE-MD	FWE-FA	FWE-MD	FWE-FA	FWE-MD	FWE-FA	FWE-MD	FWE-FA	FWE-MD
	*r*	*r*	*r*	*r*	*r*	*r*	*r*	*r*	*r*	*r*
CST	0.14	− 0.25	0.12	− 0.21	0.05	− 0.06	0.00	− 0.13	0.10	0.04
ML	0.26	-0.22	0.13	− 0.30^a,b^	0.18	− 0.27^a^	0.14	− 0.15	0.24	− 0.15
LL	0.28^a^	− 0.35^a,b^	0.17	− 0.36^a,b^	0.12	− 0.27^a^	0.14	− 0.16	0.15	− 0.42^a,b^
FPT	0.10	− 0.18	0.01	− 0.19	− 0.06	− 0.03	− 0.06	− 0.05	0.11	0.02
POTPT	0.05	− 0.42^a,b^	− 0.20	− 0.32^a,b^	0.06	− 0.21	− 0.02	− 0.22	0.06	− 0.18
STT	0.16	− 0.23	0.10	− 0.29^a,b^	0.07	− 0.35^a^	0.13	− 0.13	0.09	− 0.21
SCPCT	0.14	− 0.17	0.24	− 0.30^a,b^	0.14	− 0.17	0.03	− 0.08	0.10	− 0.19
SCPSC	0.01	− 0.09	0.01	− 0.24	0.01	− 0.12	− 0.09	0.12	− 0.01	− 0.11
ICPMC	0.11	− 0.20	0.11	− 0.39^a,b^	0.08	− 0.29^a^	0.07	− 0.05	0.13	− 0.21
ICPVC	0.15	− 0.19	0.14	− 0.40^a,b^	0.10	− 0.28^a^	0.07	− 0.09	0.06	− 0.20
MCP	0.32^a^	− 0.29^a^	0.40^a,b^	− 0.33^a,b^	0.43^a,b^	− 0.16	0.13	− 0.09	0.24	− 0.20

Partial correlations account for age, sex and average head motion during DWI brain scan

*ML* Medial lemniscus, *LL* Lateral lemniscus. *STT* Spinothalamic tract, *SCPCT* Superior cerebellar peduncle cerebellothalamic tract, *SCPSC* Superior cerebellar peduncle spinocerebellar tract, *MCP* Middle cerebellar peduncle, *ICPMC* Inferior cerebellar peduncle tracts from medulla oblongata to the cerebellum, *ICPVCT* Inferior cerebellar peduncle vestibulocerebellar tract, *FPT* Frontopontine tract, *POTPT* Parieto-occipito-temporo-pontine tract, *CST* Corticospinal tract

^a^Uncorrected *p* < .05

^b^False discovery rate corrected (FDR) *p* < .05

### Follow-Up ROI analyses with traditional (non-FWE) FA and MD

Additional follow-up analyses investigated the relationship between sensory features and brainstem white matter metrics (FA and MD) estimated using traditional tensor fitting algorithms without FWE correction. No significant relationships were found between traditionally calculated tensor metrics and sensory features after FDR correction (Additional file Table 3).

### Follow-up whole-brain VBA FWE-MD results across groups and within the autistic group

**Fig. 4:**
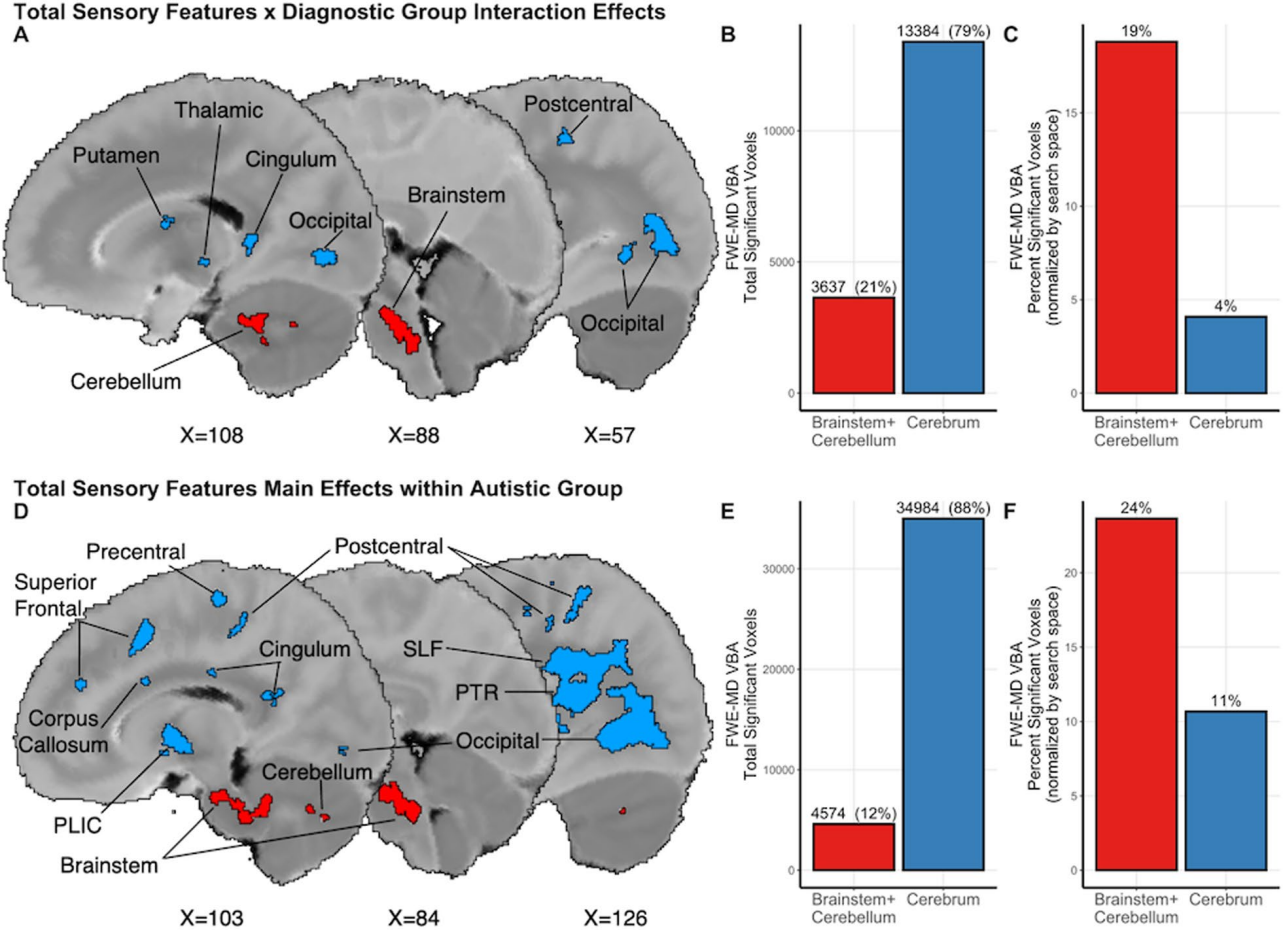
Regions with distinct sensory-microstructure relationships from whole-brain voxel-based analyses with autistic and non-autistic children. Brainstem + cerebellar white matter (red) and cerebral white matter (blue) voxels indicating a significant total sensory feature by diagnostic group interaction effects represented **A** spatially, **B** as a total count, and **C** normalized for search space (the number of possible voxels that could be found to be significantly associated with sensory features within each area). Voxels indicating a significant total sensory feature man effects within the autistic group represented **D** spatially, **E** as a total count, and **F** normalized for search space

While numerous relationships between sensory features and brainstem FWE-MD were detected, it was unclear whether these relationships were specific to the brainstem or representative of a global white matter relationship. To investigate this possibility, we conducted follow-up whole brain white matter voxel-based analyses that examined FWE-MD as function of overall sensory features (main effects) and diagnostic group-by-sensory interactions (*p* < 0.05, FDR-corrected). There were no significant main effects (i.e., cross-diagnostic sensory relations), but there were numerous, large-sized interaction clusters in the white matter of the brainstem pons, cerebellum, occipital lobe, postcentral gyrus, putamen, thalamus, and posterior cingulum (Fig. 4A, Additional file 1: Table 3). Although brainstem and cerebellar white matter comprised only 7% of the total white matter examined, 21% of the total FWE-MD voxels (3,637 mm^3^) with significant diagnostic group-by-sensory features interaction effects were in the brainstem/cerebellar white matter (Fig. 4B, Additional file 1: Table 4). When brainstem and cerebrum findings were normalized for search space (i.e., the number of possible voxels that could be found to be significantly associated with sensory features within each area), we found that 19% of the brainstem was significant, whereas only 4% of the cerebrum was significant (Fig. 4C). In all cases, FWE-MD was negatively associated with sensory features in the autistic group and positively associated with sensory features in the non-autistic group. Within the autistic group, there were numerous, large-sized main-effect clusters in the white matter of the brainstem midbrain, brainstem pons, cerebellum, occipital lobe, superior longitudinal fasciculus in the inferior parietal lobe, superior frontal lobe, precentral and postcentral gyri, posterior limb of the internal capsule, posterior thalamic radiation, corpus callosum, and cingulum (Fig. 4D, Additional file 1: Table 5). Of these, 12% of voxels that showed a significant negative relationship between sensory features and FWE-MD (4,574 mm^3^) were found in clusters within the brainstem (Fig. 4E). When brainstem and cerebrum findings were normalized for search space, 24% of the brainstem was significant, whereas only 11% of the cerebrum was significant (Fig. 4F). Taken together, these results indicate that in autistic individuals, brainstem white matter is associated with sensory features to a greater extent than would be expected based on search space alone, making it a key area of interest in understanding sensory-brain relationships in autism.

## Discussion

This study set out to identify the relationships between sensory features and white matter microstructure of the underexplored brainstem in autistic and non-autistic children. Using a novel DWI protocol that improved the apparent resolution of the brainstem and cerebellum [93], we precisely delineated brainstem and brainstem-cerebellar white matter tracts and examined their associations with total sensory features and specific sensory responses. Consistent with our hypotheses, results revealed that the microstructural properties of brainstem white matter tracts were associated with sensory features, particularly in autistic children. Together, with previous animal literature [23–28], this finding suggests that brainstem white matter contributions are not limited to relaying and processing basic sensory information, but that they extend into producing heightened or reduced sensory responses in autistic children. A follow-up whole-brain analysis demonstrated proportionally more of the sensory-brain relationships in autism occurred in the brainstem and cerebellar white matter than what we would have expected based on the size of the search area. These brainstem/cerebellar findings were contextualized by additional brain-sensory findings in white matter areas of the visual cortex, inferior parietal cortex, primary motor and sensory cortices, and thalamic radiations, all areas known to be associated with sensorimotor processing. Further, in autistic children, sensory hyporesponsiveness and tactile sensitivities were associated with white matter microstructure in nearly all brainstem tracts. These findings and their implications are discussed below.

Our study findings suggest that the brainstem plays a role in autistic children's behavioral responses to sensory stimuli. These relationships between brainstem white matter microstructure and sensory features were diagnosis-dependent and extend previous exploratory findings [42] of inverse relationships between sensory features and brain microstructure in autistic children compared to non-autistic children. These results offer intriguing insights into the potential biology underlying microstructural development of the brainstem in autism. In both the current and previous [42] studies, lower MD in the MCP and SCP were associated with more severe sensory features in autistic children but not in non-autistic children. Yet, developmental trajectories of the MCP and SCP from previous work appear to be similar in autistic and non-autistic children, with both diagnostic groups showing similar decreases in MD with age [94]. Together, this information suggests a potentially altered mechanism for sensory responsiveness in autism that heavily depends on brainstem white matter. Specifically, while lower MD is commonly interpreted as indicative of more developed (i.e., more dense and more organized) white matter tracts, present findings suggest that lower MD of the brainstem, cerebellum, and other cerebral areas, may relate to more prevalent sensory features in autistic children. This autism-specific relationship may be indicative of increased brainstem involvement in sensory responsiveness in autistic youth. It may also suggest that higher efficiency information transfer among brainstem sensory processing nuclei can lead to more prominent sensory features in autistic youth. However, MD is an indirect measure of microstructural organization and can be influenced by multiple biological factors [95]. Therefore, further research is needed to determine the precise cytoarchitectural basis of these brainstem-based relationships, using innovative and complementary quantitative MRI strategies [96, 97] that provide additional information about cellular properties of white matter.

The moderate-sized relationships between hyporesponsiveness, defined as a reduced behavioral response to stimuli in the environment, and multiple brainstem structures have implications for how we conceptualize and support diverse sensory features in autistic children. The distinct brainstem-hyporesponsiveness relationships in the autistic compared to the non-autistic groups suggest that: 1) hyporesponsiveness in non-autistic children may be neurobiologically distinct from hyporesponsiveness in autistic children in ways that current behavioral measures may not distinguish, or 2) hyporesponsiveness in autistic and non-autistic children may be an example of multifinality, in which differing neurobiological etiologies lead to similar behavioral symptoms. In either scenario, the associations among brainstem microstructural features and hyporesponsiveness in autism underscore the reflex-like orienting of hyporesponsiveness [44] and help to recontextualize the self-reports of autistic individuals [98, 99] where behavioral responses to sensory stimuli are reported to feel outside of volitional control. Therefore, therapies that use external reward or punishment to target sensory features may be unlikely to be successful as they assume volitional control and are unlikely to target the brainstem-based neural circuitry that may underlie sensory hyporesponsiveness in autistic individuals. Previous research demonstrated that a six-week biofeedback-based training in autistic and non-autistic adolescents induced treatment-specific changes to the superior cerebellar peduncle [88], a region found to be associated with sensory features in both the present study and Wolff et al*.* [42] Therefore, there is preliminary evidence of brainstem microstructural changes in response to a multi-week intervention. Used in the context of sensory interventions, future studies should track brainstem changes in relation to intervention-related decreases in sensory features.

The present findings also suggest that brainstem white matter may be particularly related to tactile responsivity in autistic individuals, with eight of the 11 brainstem tracts moderately related to responses to touch. Tactile sensitivity has been commonly reported in autistic individuals [100–103], and reduced tactile responsivity at 12 months was found to be an early predictor of a later autism diagnosis [104]. Furthermore, the inferior olivary nucleus (ION) in the upper medulla aspect of the brainstem is associated with integration of tactile sensations with motor responses and has been previously found to have atypical structure in postmortem brain analysis of autistic individuals [105–107]. The ION receives numerous brainstem and cerebellar inputs (as reviewed in [18]) and outputs to the cerebellum via portions of the inferior cerebellar peduncle. Therefore, it is possible that the early-developing brainstem is implicated in tactile experiences of autistic school-aged children in ways that involve the ION. However, future research will be needed to confirm and further examine this relationship, particularly given that the present sensory measure cannot disentangle pain, pressure, and vibration. Fortunately, enhanced imaging of the brainstem may enable elucidation of the size, shape, and microstructural properties of specific brainstem nuclei, like the ION, in future in vivo studies of autistic children and adults.

The follow-up whole-brain analyses further contextualized the present sensory-brainstem findings, by showing that sensory features in autistic children were also related to cerebral white matter in brain areas frequently associated with sensory processing, including the occipital cortex (vision), inferior parietal cortex (audition), primary motor and somatosensory cortices (touch and proprioception), and thalamic projections (multisensory relay). One interpretation of these results is that the brainstem findings are reflective of a whole-brain sensory phenomenon, whereby decreased mean diffusivity is related to more sensory features in autistic children. However, our results also suggested that brainstem and cerebellum findings are overrepresented with respect to the size of the search space, suggesting that the brainstem and cerebellar white matter tracts may play a strong role in the sensory experiences of autistic individuals. These findings are compatible with the brainstem’s involvement in prenatal development of the cortex ([108–110]) and the cascading effects on the brain that prenatal brainstem differences combined with ongoing sensorimotor tuning may have [18, 20]. However, longitudinal studies, ideally from early prenatal development into the first few years of postnatal development, will be needed to determine the exact role of the brainstem and cerebellum in sensory processing and overall brain development. In all, the present findings, combined with theoretical work and studies implicating the brainstem in autism [18–20], suggest that the brainstem and cerebellum may be integral contributors to the sensory experiences of autistic individuals. Therefore, even though the imaging of the brainstem may require special acquisition and processing procedures [49], including free water elimination, EPI distortion correction, and careful consideration of brainstem masking, these steps are worth taking, as the brainstem and cerebellum are likely key areas to study to better understand the neurobiological basis of the autistic experience.

## Limitations

The present findings should be interpreted considering study strengths and limitations. Due to COVID-19 restrictions on in-person research, our sample size in the group of autistic participants was below that which we had intended by a conservative a priori power analysis. However, the present sample size is still one of the largest in the literature. Future research will be needed to replicate these findings. While consistent with the 5–15% of non-autistic children in the general population who exhibit elevated sensory features, a notable limitation was the proportionally small number of participants in the non-autistic group with elevated sensory symptoms, which may have constrained detection of the neural correlates of sensory responsivity in the non-autistic group. Future studies contrasting a sensory processing disorder cohort with autistic individuals in this age range are warranted. Further, our measure of sensory features was limited to caregiver report. Based on evidence suggesting neurobiological relations with observed sensory measures [34], it is possible that even clearer relationships may emerge in combination with observed measures, which will be a key avenue for future research. Moreover, our analyses only analyzed one sensory pattern at a time even though sensory patterns often co-occur [111]. Future research should examine combinations of sensory patterns. Finally, all participants in this study communicated with our study team verbally and were able to acclimate to the sensory environment of an MRI session, and it is possible that children requiring higher cognitive support or sensory responsivity may have opted out of participating which should be considered when assessing the generalizability of our findings to the whole of the autism spectrum.

## Conclusions

In summary, the present study evaluated the relationships between brainstem white matter microstructure and sensory features in autistic and non-autistic children. The findings revealed distinct white matter underpinnings of elevated sensory features in autistic children compared to non-autistic children that were prominent in the brainstem and suggestive of a distinct etiology of sensory features in autism. Hyporesponsiveness and tactile responsivity were associated with numerous brainstem tracts in autistic children, suggesting the early-developing and reflex-like nature of sensory orienting and tactile responses in autism. These findings are among the first to suggest that sensory features are aligned with white matter microstructure of the brainstem and support the theory of unique brainstem contributions to behavior in autistic individuals.

## Supplementary Information

**Additional file 1.** Supplementary information and data analyses.

## Data Availability

A portion of these data are openly available in National Institute of Mental Health Data Archive at http://doi.org/10.15154/1523353, reference number 3088. The remaining data that support the findings of this study are available from the corresponding author, upon reasonable request.

## Abbreviations

DWI: Diffusion-weighted imaging
DTI: Diffusion tensor imaging
FWE: Free water eliminated
FA: Fractional anisotropy
MD: Mean diffusivity
WASI-2: Wechsler abbreviated scale of intelligence, 2nd edition
KBIT-2: Kaufman brief intelligence test-second edition
ADOS-2: Autism diagnostic observation schedule, 2nd edition
ADI-R: Autism diagnostic interview-revised
SCQ: Social communication questionnaire
AVD: Average volume displacement
SEQ 3.0: Sensory experience questionnaire
EPI: Echo planar imaging
SCPCR: Superior cerebellar peduncle-cerebellorubral
SCPCT: Superior cerebellar peduncle-cerebellothalamic
ROI: Region of interest
MCP: Middle cerebellar peduncle
FDR: False discovery rate
T-SPOON: Tissue-specific, smoothing-compensated
VBA: Voxel-based analysis
PALM: Permutation analysis of linear models
CST: Corticospinal tract
ML: Medial lemniscus
FPT: Frontopontine tract
POTPT: Parieto-occipito-temporo-pontine tract
ASD: Autism spectrum disorder
