# Supplementary material for: Brainstem white matter microstructure is associated with hyporesponsiveness and overall sensory features in autistic children
Source: Mol Autism. 2022 Dec 19;13:48. doi: 10.1186/s13229-022-00524-3 (PMC9762648; doi:10.1186/s13229-022-00524-3)
Supplement: Supplementary file 1 — Additional file 1. Supplementary information and data analyses. [file 13229_2022_524_MOESM1_ESM.pdf]

Supplementary Materials for

Brainstem White Matter Microstructure is Associated with Hyporesponsiveness and Overall Sensory Features in Autistic Children

Authors: Olivia Surgent[1,2], Ali Riaz[1], Karla K. Ausderau[1,3], Nagesh Adluru[1,4], Gregory R. Kirk[1], Jose Guerrero-Gonzalez[1,5], Emily C. Skaletski[1,3], Steven R. Kecskemeti[1], Douglas C Dean III[1,5,6], Susan Ellis Weismer[1,7,8,9], Andrew L. Alexander[1,5,10], Brittany G. Travers[1,3]

Author Affiliations: [1]Waisman Center, University of Wisconsin-Madison, Madison, WI, USA , [2]Neuroscience Training Program, University of Wisconsin-Madison, Madison, WI, USA, [3]Occupational Therapy Program in the Department of Kinesiology, University of Wisconsin-Madison, Madison, WI, USA, [4]Department of Radiology, University of Wisconsin-Madison, Madison, WI, USA, [5]Department of Medical Physics, University of Wisconsin-Madison, Madison, WI, USA, [6]Department of Pediatrics, University of Wisconsin-Madison, Madison, WI, USA, [7]Department of Communication Sciences and Disorders, University of Wisconsin-Madison, Madison, WI, USA, [8]Department of Psychology, University of Wisconsin-Madison, Madison, WI, USA, [9]Department of Educational Psychology, University of Wisconsin-Madison, Madison, WI, USA, [10]Department of Psychiatry, University of Wisconsin-Madison, Madison, WI, USA

Author Email Addresses: Olivia Surgent: osurgent@wisc.edu, Ali Riaz: aariaz@wisc.edu, Karla Ausderau: kausderau@wisc.edu, Nagesh Adluru: adluru@wisc.edu, Gregory Kirk: gkirk@wisc.edu, Jose Guerrero-Gonzalez: jguerrerogon@wisc.edu, Emily C Skaletski: eskaletski@wisc.edu, Steven R. Kecskemeti: kecskemeti@wisc.edu, Doug Dean III: deaniii@wisc.edu, Susan Ellis Weismer: susan.ellisweismer@wisc.edu, Andrew Alexander: andy.alexander@wisc.edu, Brittany G Travers: btravers@wisc.edu

Corresponding author's contact information:
Brittany G. Travers
Waisman Center
University of Wisconsin-Madison
1500 Highland Avenue, Room 435
Madison, WI 53705
Phone: (608) 263-0282
Fax: (608) 263-0529
Email: btravers@wisc.edu

Additional Methods: Power analysis. The effects that require the most statistical power in this study are the voxel-based analysis interaction effects. A conservative *a priori* power estimated that 71 participants in each group would provide 90% power to detect interaction effects of interest in our voxel-based analysis. However, this was a very conservative estimate (based on the challenges of calculating power for an analysis like this that additionally uses an *fdr* correction). Due to the impact of the COVID-19 pandemic on our ability to run participants in-person, our final sample size of complete data was 63 autistic participants and 74 non-autistic participants. Rigorous quality control checks additionally flagged 2 DWI scans in the non-autistic group and 1 DWI scan in the autistic group to not include. Additionally, we found 1 SEQ score in the autistic group to have outlying values that affected that analysis, and they were therefore omitted. This left a final sample size of 61 autistic participants and 72 non-autistic participants.

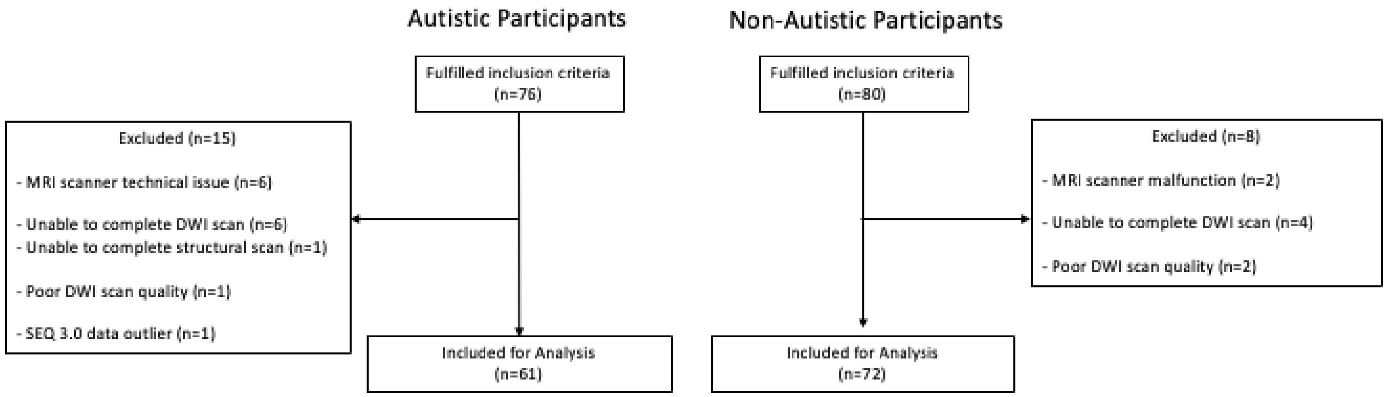

Supplementary Figure 1. Study enrollment flow chart. Information about potential participants who were screened for inclusion but did not meet inclusion criteria was not recorded and therefore is not included. The scanner malfunction was due to an upgrade that changed the scan parameters during a time in which 8 participants were scanned. Rigorous quality control checks of the diffusion weighted imaging (DWI) data determined poorer data quality in 3 participants. Therefore, these 3 participants were excluded from analyses. Examination of the caregiver reported Sensory Experience Questionnaire 3.0 (SEQ 3.0) data identified 1 extreme outlier within the autistic group (Cook's distances >4/*n*). This participant was therefore excluded from the analyses.

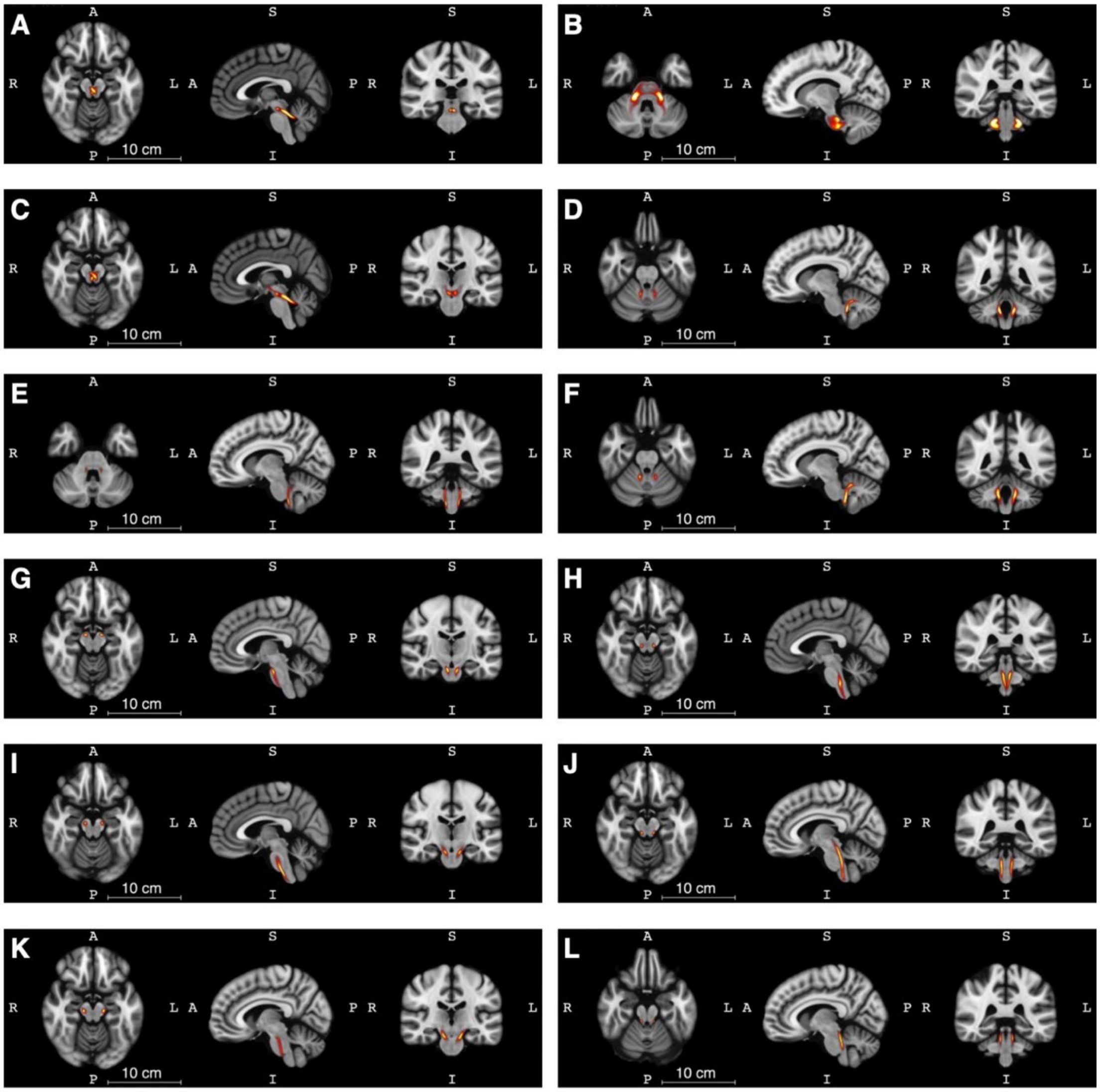

Supplementary Figure 2. Bilateral brainstem white matter bundles from the probabilistic brainstem connectome atlas [19] visualized with MNI 152 T1 underlay. (A) Superior cerebellar peduncle cerebellorubral tract (SCPCR), (B) middle cerebellar peduncle (MCP), (C) superior cerebellar peduncle cerebellothalamic tract (SCPCT), (D) superior cerebellar peduncle spinocerebellar tract (SCPSC), (E) inferior cerebellar peduncle vestibulocerebellar tract (ICPVC), (F) inferior cerebellar peduncle medulla-cerebellar tract (ICPMC), (G) fronto-pontine tract (FPT), (H) medial lemniscus (ML), (I) corticospinal tract (CST), (J) spinothalamic tract (STT), (K) parieto-occipito-temporo-pontine tract (POTPT), (L) lateral lemniscus (LL).

Supplementary Table 1. Autistic vs non-autistic group differences in brainstem FWE-DTI metrics

Brainstem White Matter Region	FWE-FA				FWE-MD			
	b	Std Error	t	p	b	Std Error	t	p
CST	0.005	0.007	0.72	.47	-0.001	0.003	-0.28	.78
ML	0.003	0.005	0.58	.56	-0.005	0.002	-1.89	.06
LL	0.010	0.008	1.26	.21	-0.005	0.003	-1.56	.12
FPT	0.012	0.008	1.54	.13	-0.001	0.004	-0.23	.82
POTPT	0.005	0.006	0.85	.40	0.000	0.003	-0.15	.88
STT	0.006	0.005	1.20	.23	-0.004	0.002	-1.76	.08
SCPCT	-0.001	0.004	-0.15	.88	-0.002	0.002	-0.66	.51
SCPSC	0.011	0.006	1.74	.08	-0.006	0.003	-1.58	.12
ICPMC	0.011	0.006	2.01	.05	0.001	0.003	0.23	.82
ICPVC	0.012	0.006	1.93	.06	0.001	0.003	0.27	.79
MCP	0.006	0.005	1.13	.26	-0.002	0.003	-0.68	.50

Group difference effects controlling for age, sex and average head motion during DWI brain scan

ML medial lemniscus; LL lateral lemniscus; STT spinothalamic tract; SCPCT superior cerebellar peduncle cerebellothalamic tract; SCPSC superior cerebellar peduncle spinocerebellar tract; MCP middle cerebellar peduncle; ICPMC inferior cerebellar peduncle tracts from medulla oblongata to the cerebellum; ICPVCT inferior cerebellar peduncle vestibulocerebellar tract; FPT frontopontine tract; POTPT parieto-occipito-temporo-pontine tract; CST corticospinal tract

a Uncorrected p<.05
b False discovery rate (FDR) corrected p<.05

Supplementary Table 2. Effects of total sensory features on brainstem traditional DTI in autistic and non-autistic children

Brainstem White Matter Region	FWE-FA								FWE-MD							
	Laterality Main Effect				Laterality x Total Sensory Feature x Group Interaction Effect				Laterality Main Effect				Laterality x Total Sensory Feature x Group Interaction Effect			
	b	Std Error	t	p	b	Std Error	t	p	b	Std Error	t	p	b	Std Error	t	p
CST	-0.005	0.023	-0.22	0.83	-0.009	0.019	-0.50	0.62	-0.00691	0.01213	-0.6	0.57	-0.00817	0.01011	-0.8	0.42
ML	-0.029	0.013	-2.20	0.03[a]	-0.026	0.011	-2.43	0.02[a]	-0.02520	0.00788	-3.2	0.01[a]	-0.00821	0.00657	-1.2	0.23
LL	0.008	0.033	0.26	0.80	0.013	0.027	0.49	0.63	0.03650	0.01848	2.0	0.05	0.00276	0.01539	0.2	0.86
FPT	0.037	0.030	1.25	0.21	0.027	0.025	1.08	0.28	-0.01475	0.01436	-1.0	0.30	-0.01104	0.01196	-0.9	0.36
POTPT	-0.049	0.025	-1.97	0.05	0.005	0.021	0.26	0.80	-0.00266	0.00859	-0.3	0.76	-0.00480	0.00715	-0.7	0.50
STT	-0.021	0.018	-1.13	0.26	-0.007	0.015	-0.45	0.66	0.00825	0.01162	0.7	0.48	-0.00859	0.00968	-0.9	0.37
SCPCT	-0.017	0.010	-1.72	0.09	0.001	0.008	0.15	0.88	0.00704	0.00595	1.2	0.25	0.01242	0.00496	2.5	0.02[a]
SCPSC	0.004	0.025	0.14	0.89	0.019	0.021	0.90	0.37	0.01058	0.01455	0.7	0.47	0.00093	0.01212	0.1	0.94
ICPMC	-0.003	0.018	-0.16	0.87	0.010	0.015	0.64	0.52	0.02094	0.01071	2.0	0.05	0.00843	0.00892	0.9	0.35
ICPVC	-0.014	0.018	-0.77	0.44	-0.012	0.015	-0.78	0.44	0.00911	0.01026	0.9	0.37	0.01076	0.00855	1.3	0.21

Main and interaction effects controlling for age, sex and average head motion during DWI brain scan

ML medial lemniscus; LL lateral lemniscus; STT spinothalamic tract; SCPCT superior cerebellar peduncle cerebellothalamic tract; SCPSC superior cerebellar peduncle spinocerebellar tract; ICPMC inferior cerebellar peduncle tracts from medulla oblongata to the cerebellum; ICPVCT inferior cerebellar peduncle vestibulocerebellar tract; FPT frontopontine tract; POTPT parieto-occipito-temporo-pontine tract; CST corticospinal tract

a Uncorrected p<.05
b False discovery rate (FDR) corrected p<.05

Supplementary Table 3. Effects of total sensory features on brainstem traditional DTI in autistic and non-autistic children

Brainstem White Matter Region	FWE-FA								FWE-MD							
	Total Sensory Features Main Effect				Group x Total Sensory Features Interaction Effect				Total Sensory Features Main Effect				Group x Total Sensory Features Interaction Effect			
	b	Std Error	t	p	b	Std Error	t	p	b	Std Error	t	p	b	Std Error	t	p
CST	-0.002	0.010	-0.20	.84	0.007	0.020	0.35	.72	0.007	0.006	1.25	.21	-0.013	0.011	-1.12	.26
ML	-0.006	0.008	-0.81	.42	0.027	0.016	1.71	.09	0.000	0.004	-0.06	.95	-0.001	0.009	-0.16	.87
LL	0.009	0.012	0.80	.43	0.011	0.024	0.44	.66	-0.012	0.011	-1.11	.27	0.025	0.023	1.12	.27
FPT	-0.023	0.012	-2.01	.05	0.035	0.024	1.44	.15	0.018	0.007	2.38	.02[a]	-0.039	0.015	-2.54	.01[a]
POTPT	-0.010	0.008	-1.27	.21	0.006	0.017	0.36	.72	0.002	0.005	0.42	.67	-0.010	0.010	-1.00	.32
STT	0.007	0.007	0.91	.37	0.000	0.015	0.00	1.00	-0.001	0.005	-0.11	.91	0.002	0.009	0.23	.82
SCPCT	0.001	0.005	0.23	.82	0.004	0.011	0.37	.71	0.000	0.004	0.08	.94	-0.008	0.008	-1.01	.32
SCPSC	-0.008	0.008	-1.02	.31	0.006	0.017	0.39	.70	-0.003	0.009	-0.30	.76	0.011	0.018	0.60	.55
ICPMC	0.002	0.008	0.29	.77	0.011	0.017	0.66	.51	-0.001	0.006	-0.12	.90	-0.018	0.012	-1.51	.13
ICPVC	0.007	0.009	0.76	.45	0.007	0.018	0.40	.69	0.000	0.006	0.03	.97	-0.016	0.012	-1.34	.18
MCP	0.009	0.008	1.19	.23	0.016	0.016	1.02	.31	0.004	0.004	0.89	.38	-0.016	0.009	-1.76	.08

Main and interaction effects controlling for age, sex and average head motion during DWI brain scan

ML medial lemniscus; LL lateral lemniscus; STT spinothalamic tract; SCPCT superior cerebellar peduncle cerebellothalamic tract; SCPSC superior cerebellar peduncle spinocerebellar tract; MCP middle cerebellar peduncle; ICPMC inferior cerebellar peduncle tracts from medulla oblongata to the cerebellum; ICPVCT inferior cerebellar peduncle vestibulocerebellar tract; FPT frontopontine tract; POTPT parieto-occipito-temporo-pontine tract; CST corticospinal tract

a Uncorrected p<.05
b False discovery rate (FDR) corrected p<.05

Supplementary Table 4. FWE-MD clusters representing total sensory features-by-diagnostic group interaction effects

Region	Right/Left	Cluster Size (voxels)	X	Y	Z	p-value
Middle Occipital White Matter, Sagittal Stratum	Right	6548	52	68	70	<.001
Pontine Crossing Tract, Brainstem White Matter	Left	2600	88	101	42	<.001
Inferior Occipital White Matter	Left	1424	122	46	70	<.001
Putamen	Right	1072	66	124	87	<.001
Inferior Cerebellar Peduncle	Right	795	78	82	35	<.001
Substantia Nigra	Left	620	121	99	77	<.001
Precentral White Matter	Right	433	64	107	140	.004
Supramarginal White Matter	Right	416	57	92	90	<.001
Cingulum	Right	390	81	80	93	<.001
Posterior Thalamic Radiation	Left	367	118	62	71	<.001
Thalamus	Right	359	70	102	69	.002
Superior Parietal Lobule	Right	318	54	86	122	<.001
Postcentral White Matter	Right	272	59	94	123	.008
Cuneus White Matter	Left	212	104	32	83	<.001
Inferior Cerebellar Peduncle	Left	185	103	83	40	<.001
Supramarginal White Matter	Right	162	36	91	90	<.001
Angular White Matter	Right	116	48	58	108	.017
Precentral White Matter	Right	115	34	129	82	<.001
Postcentral White Matter	Left	90	124	95	121	.003
Superior Parietal White Matter	Left	77	118	81	126	.041
Postcentral White Matter	Right	55	46	100	128	.004
Cingulum White Matter	Right	53	78	71	99	.036
Middle Occipital Gyrus	Left	50	103	40	62	.014
Middle Temporal White Matter	Right	44	51	75	87	.021
Superior Parietal White Matter	Left	37	106	59	106	.030
Cerebellar White Matter	Right	28	74	62	39	.023
Sagittal Stratum	Left	22	127	83	60	.025
Superior Temporal White Matter	Right	17	48	99	71	.016
Middle Temporal White Matter	Right	17	47	86	76	.033
Inferior Temporal White Matter	Right	17	37	81	62	.007
Superior Temporal White Matter	Right	15	48	91	71	.026
Postcentral White Matter	Left	14	129	93	130	.041
Postcentral White Matter	Left	12	127	101	115	.044
Middle Cerebellar Peduncle	Left	12	103	102	43	.006
Superior Parietal White Matter	Left	11	110	66	101	.041
Corticospinal Tract	Left	9	95	99	34	.034
Postcentral White Matter	Left	9	135	107	124	.018
Cingulum	Right	5	83	106	107	.047
Cerebellar White Matter	Right	4	84	68	54	.045
Postcentral White Matter	Left	3	128	94	128	.047
Superior Cerebellar peduncle	Right	3	82	86	40	.035
Superior Parietal White Matter	Left	2	99	57	124	.044
Cingulum White Matter	Left	2	98	70	92	.049
Superior Temporal White Matter	Left	2	137	101	71	.044
Cingulum	Right	1	82	86	42	.045
Corpus Callosum (Body)	Right	1	80	108	100	.048
Angular White Matter	Right	1	53	86	99	.048
Superior Corona Radiata	Right	1	69	105	93	.046
Cingulum	Right	1	69	90	66	.049
Middle Occipital White Matter	Right	1	69	37	75	.048
Midbrain	Left	1	97	101	60	.033

Supplementary Table 5. FWE-MD clusters representing main effects of total sensory features in autistic group

Region	Right/Left	Cluster Size (voxels)	X	Y	Z	p-value
Superior Temporal White Matter, Superior Longitudinal Fasciculus	Right	21082	54	65	66	<.001
Pontine Crossing Tract	Left	4206	93	90	32	<.001
External Capsule Superior Corona Radiata	Right	2069	65	129	90	<.001
Superior Frontal White Matter	Right	1491	75	142	120	<.001
Posterior Thalamic Radiation	Left	1173	120	61	72	<.001
Supramarginal White Matter	Left	954	129	88	96	<.001
Middle Frontal White Matter	Left	896	117	142	110	<.001
Superior Parietal White Matter	Right	630	58	83	130	<.001
Posterior Limb of Internal Capsule	Right	533	80	124	72	<.001
Cuneus White Matter	Left	525	109	34	88	.001
Superior Frontal White Matter	Right	481	70	123	124	<.001
External Capsule	Right	434	60	138	77	<.001
Superior Frontal White Matter	Right	419	74	168	99	.004
Inferior Frontal White Matter	Right	385	54	157	81	.023
Precentral White Matter	Left	345	117	105	116	.027
Middle Frontal Orbital White Matter	Right	345	71	146	57	.001
Inferior Frontal White Matter	Right	310	56	136	87	.005
Anterior Corona Radiata	Left	301	103	163	82	<.001
Superior Parietal White Matter	Left	262	116	82	124	.015
Anterior Corona Radiata	Left	254	106	152	98	.011
Cingulum (Cingulate Gyrus)	Right	193	82	106	104	.014
Precentral White Matter	Right	171	75	103	142	.031
Superior Temporal White Matter	Left	166	119	102	89	.018
Middle Frontal White Matter	Left	143	114	162	109	<.001
Middle Frontal White Matter	Right	143	58	137	126	.006
Middle Cerebcellar peduncle	Right	136	65	70	29	<.001
Superior Frontal White Matter	Left	123	107	181	90	.001
Superior Frontal White Matter	Left	113	109	127	138	<.001
Cingulum	Right	112	72	85	77	.034
Cingulum	Left	89	102	79	79	.038
Posterior Corona Radiata	Left	69	112	103	97	.036
Globus Pallidus	Right	69	74	128	66	<.001
Corpus Callosum (Body)	Right	60	77	139	101	.037
Cerebellar White Matter	Left	60	102	50	35	.015
Middle Frontal White Matter	Right	57	57	179	76	.032
Cerebellar White Matter	Left	55	106	63	38	.014
Cerebellar White Matter	Right	55	73	54	32	<.001
Lateral Fronto Orbital White Matter	Right	54	64	153	60	.014
Middle Frontal White Matter	Right	54	56	145	119	.036
Middle Frontal White Matter	Left	49	122	158	94	.037
Superior Corona Radiata	Left	41	122	110	107	.034
Postcentral White Matter	Right	38	51	107	114	.003
Inferior Frontal White Matter	Right	31	47	165	67	.033
Caudate Nucleus	Left	31	108	118	92	.038
Insular White Matter	Right	28	62	147	82	.032
Precentral White Matter	Left	27	106	99	123	.038
Inferior Frontal White Matter	Right	27	38	144	83	.031
Superior Corona Radiata	Right	26	61	124	110	.029
Fornix Stria Terminalis	Right	26	70	94	80	.033
Postcentral White Matter	Right	26	52	94	135	.027
Cerebellar White Matter	Right	23	64	59	29	.001
Middle Occipital White Matter	Left	18	120	50	77	.045

Cerebellar White Matter	Right	16	55	64	34	.035
Posterior Corona Radiata	Left	15	117	79	97	.036
Middle Occipital Gyrus	Right	11	65	38	92	.042
Cingulum	Right	10	83	148	96	.035
Superior Frontal White Matter	Left	10	101	167	113	.038
Inferior Frontal White Matter	Left	10	121	171	79	.044
Medulla	Right	9	90	88	12	.023
Cerebellar White Matter	Right	8	75	76	29	.001
Precentral White Matter	Left	6	145	131	79	.031
Middle Frontal White Matter	Left	5	111	168	110	.036
Anterior corona radiata	Right	5	69	155	101	.048
Inferior Fronto Occpital Fasciculus	Left	4	110	142	56	.033
Insular White Matter	Left	4	114	145	54	.044
Middle Occipital White Matter	Left	4	125	54	77	.048
Superior Temporal White Matter	Right	3	37	111	70	.049
Superior Frontal Gyrus	Left	3	103	189	74	.046
Inferior Frontal White Matter	Right	3	52	158	65	.049
Superior Frontal White Matter	Right	2	79	157	125	.047
Cerebellar White Matter	Right	2	84	68	54	.048
Superior Frontal White Matter	Right	2	75	162	111	.050
Superior Frontal White Matter	Right	2	76	150	110	.050
Cerebellar White Matter	Left	2	107	71	42	.045
Cerebellar White Matter	Right	2	59	61	35	.041
Supramarginal White Matter	Right	2	34	89	103	.043
Cerebellar White Matter	Left	1	98	62	43	.046
Cingulum	Right	1	82	146	97	.044
Superior Parietal White Matter	Left	1	114	87	117	.049
Fornix	Left	1	119	112	62	.046
Superior Frontal White Matter	Left	1	105	188	76	.047
Cerebellar White Matter	Left	1	116	81	27	.048
Superior Corona Radiata	Right	1	67	128	99	.049
Superior Frontal White Matter	Right	1	80	157	123	.048
Thalamic White Matter	Right	1	66	105	73	.049
Inferior Frontal White Matter	Right	1	50	164	71	.047
